# Biophysical Insights into Cancer Transformation and Treatment

**DOI:** 10.1155/2013/195028

**Published:** 2013-06-11

**Authors:** Jiří Pokorný, Alberto Foletti, Jitka Kobilková, Anna Jandová, Jan Vrba, Jan Vrba, Martina Nedbalová, Aleš Čoček, Andrea Danani, Jack A. Tuszyński

**Affiliations:** ^1^Institute of Photonics and Electronics, Academy of Sciences of the Czech Republic, AS CR, Chaberská 57, 182 51 Prague 8-Kobylisy, Czech Republic; ^2^Institute of Translational Pharmacology, National Research Council-CNR, Via Fosso del Cavaliere 100, 00133 Rome, Italy; ^3^University of Applied Sciences of Southern Switzerland-SUPSI, Department of Innovative Technologies, Galleria 2, 6928 Manno, Switzerland; ^4^1st Faculty of Medicine, Charles University in Prague, Department of Obstetrics and Gynaecology, Apolinářská 18, 128 00 Prague 2, Czech Republic; ^5^Faculty of Electrical Engineering, Czech Technical University in Prague, Technická 2, 166 27 Prague 6, Czech Republic; ^6^Faculty of Biomedical Engineering, Czech Technical University in Kladno, Sitná Square 3105, 272 01 Kladno, Czech Republic; ^7^1st Faculty of Medicine, Charles University in Prague, Institute of Physiology, Albertov 5, 128 00 Prague 2, Czech Republic; ^8^3rd Faculty of Medicine, Charles University in Prague, Department of Otorhinolaryngology, Ruská 87, 100 00 Prague 10, Czech Republic; ^9^Department of Physics, University of Alberta, Edmonton, AB, Canada T6G 2J7

## Abstract

Biological systems are hierarchically self-organized complex structures characterized by nonlinear interactions. Biochemical energy is transformed into work of physical forces required for various biological functions. We postulate that energy transduction depends on endogenous electrodynamic fields generated by microtubules. Microtubules and mitochondria colocalize in cells with microtubules providing tracks for mitochondrial movement. Besides energy transformation, mitochondria form a spatially distributed proton charge layer and a resultant strong static electric field, which causes water ordering in the surrounding cytosol. These effects create conditions for generation of coherent electrodynamic field. The metabolic energy transduction pathways are strongly affected in cancers. Mitochondrial dysfunction in cancer cells (Warburg effect) or in fibroblasts associated with cancer cells (reverse Warburg effect) results in decreased or increased power of the generated electromagnetic field, respectively, and shifted and rebuilt frequency spectra. Disturbed electrodynamic interaction forces between cancer and healthy cells may favor local invasion and metastasis. A therapeutic strategy of targeting dysfunctional mitochondria for restoration of their physiological functions makes it possible to switch on the natural apoptotic pathway blocked in cancer transformed cells. Experience with dichloroacetate in cancer treatment and reestablishment of the healthy state may help in the development of novel effective drugs aimed at the mitochondrial function.

## 1. Introduction

Partial suppression of oxidative metabolism in cancers was first discovered by Warburg et al. [1]. He proved its origin in the diminished activity of mitochondria [2]. This was an extraordinary insight that took half a century for the scientific community to be fully appreciated. It is worth noting that Warburg understood biological systems as highly organized structures at the time when little was known about the internal organization of a living cell. This led him to an intuitive conclusion that disturbances of oxidative metabolism are an essential part of cancer initiation and progression. However, at the time of Warburg's discovery, this point of view was not accepted by the scientific community regardless of its genuine significance. Contemporary comprehension of biological systems is connected with the idea of complexity (for a description of complex systems, the reader is referred, for instance, to Cohen and Havlin [3], and a simple model is developed in [4]). Biological systems are examples of complex systems composed of a large number of nonlinearly interacting elements organized into hierarchical structures [5]. These systems of interconnected entities exhibit emergent phenomena where the whole possesses properties not present in their individual parts. The organized ensemble of elements, therefore, creates new features and forms of activity. Biological systems are open since they exchange mass, energy, and information with their environments. They are dissipative structures. The whole complex of any biological system is a result of self-organization [6] under nonequilibrium thermodynamic conditions (far from thermodynamic equilibrium). Adaptability is another feature of biological systems based on interactions with their surroundings. Environmental influences lead to constant modifications of internal structure and patterns of activity. Interaction with the surroundings is not only passive but also displays active action. Branching of reaction and activity pathways is a general property of biological systems. Consequently, the knowledge regarding the composition and activity of only one component of biological systems is of limited value due to parallel interconnections.

Biological systems display a central control and steering which is provided by brain activity in mammals. The brain receives information from individual parts of the hierarchical system, processes it, and reacts to it by sending controlling signals. Body communication systems with information channels are an indispensable part of the brain's control-and-command function. However, information transfer in biological systems has been up to the present time analyzed as a transfer of quality, or an order of entities [7]. Reduction to a quantitative basis has not been performed so far, and, therefore, assessment of the amount of information or channel capacity has not been possible. Action potential propagating along nerve fibres as an information transfer medium seems to have insufficient capacity to provide a required communication function. Internal cooperation and coherent activity in mammalian species require high capacity information transfer between the central control unit—the brain—and the periphery—the organs. Photon information transfer was argued to be essential in biology in general [8]. Importantly, recent results show the ability of cancer cells to cause injury in distant healthy tissues by a physical mechanism of information transfer [9]. The changes in the tissue are possibly triggered by biophotons emitted by cancer cells. Biophotons may propagate through the soft tissues, inside nerve fibers, and inside or along other structures used as conduits in the body. Notably, biophoton transfer along the nerves of rats has been demonstrated experimentally [10]. Disturbances of the information transferred to and from the brain in a pathological cancer state have not been demonstrated yet.

Mitochondrial dysfunction in cancer cells was considered as an unimportant effect at Warburg's time and even long after his death. Biological research was primarily based on the examination of morphology, composition, chemical reactions, and information transfer by mass elements. Physical processes in biological systems were not accepted as an essential part of living activity. Subsequently, Fröhlich proposed that coherent electrical polar oscillations and the generation of electromagnetic fields play important roles in living cells [11–15], and their disturbances occur in cancer cells [16]. Similarly to Warburg, Fröhlich was ahead of his time. Structures generating the electromagnetic field were not discovered at that time and nanotechnological measurement methods were not in existence yet. However, experimental support for Fröhlich's ideas was being gradually accumulated. Measurements performed on living cells disclosed electric and electromagnetic oscillations. Dielectrophoretic forces of the cellular oscillating electric field cause attraction of dielectric particles which depends on their permittivity [17]. Further measurements were performed by Hölzel and Lamprecht [18] and Hölzel [19] who proved electric origin of the forces acting on dielectric particles. The generation of cellular electromagnetic fields, however, was not ascribed to microtubules discovered by Amos and Klug [20] regardless of the extensive microtubule research at the time. However, both experimental and theoretical research of the cellular electromagnetic activity gradually pointed to microtubules as major sources of electromagnetic interactions [21, 22].

Very likely the most interesting phenomenon connected with the microtubule research is water ordering taking place in living cells. Layers of water without solutes observed around microtubules were called clear zones [23]. Formation of clear zones was assumed to depend on the negative electrostatic charge at the microtubule surface [24]. Ling formulated a theory of the ordering of water molecules in the electrostatic field of the surface charges at the interface [25]. The clear (exclusion) zones were proved to be layers of ordered water [26–28]. Interfacial water ordering may be formed up to a distance of about 0.1 mm from the charged surface. Ions are excluded from the ordered layer due to its strong electric field, thermal fluctuations are diminished as follows from the measurements in the range of wavelengths of 3.8–4.6 *μ*m, and UV absorbance at 270 nm which is increased. The ordered layer of water molecules resembles a gel. Ordered water layers are formed around mitochondria [29], which follows from the experimental results published by Tyner et al. [30].

The physical structure of water was analyzed by Preparata [31], Del Giudice et al. [32], and Del Giudice and Tedeschi [33] on the basis of the quantum electrodynamic theory. The liquid water is a mixture of two phases of water: ordered water forming coherent domains and gas-like water (bulk water). The clear (exclusion) zones display macroscopic separation of these two phases of water caused by a strong electric field.

Elastic oscillations of the yeast cell membrane in the acoustic range below 2 kHz were measured by Pelling et al. [34, 35], and elastic and electric oscillations were compared [36, 37]. Microtubule polymerization in cells may be disrupted by external electromagnetic field in the frequency range 0.1–0.3 MHz [38, 39]. Electric oscillations at cellular membrane of yeast and alga cells in the frequency range 1.5–52 MHz were measured [18, 19]. The high values of the electrodynamic activity of synchronized yeast cells in the M phase coincide with the periods of arrangement of the microtubules into a mitotic spindle, during metaphase, and anaphase A and B [22]. Damping of external electromagnetic field caused by cancer tissue at the frequency 465 MHz and the first harmonic was experimentally determined by Vedruccio and Meessen [40]. Oscillations in microtubules may be damped in cancer cells by water with decreased level of ordering [41]. Cancer cells exhibit a less-ordered structure [42]. Interactions between cells mediated by cellular electromagnetic fields in the red and near-infrared range were observed by Albrecht-Buehler [43–45].

Electromagnetic resonant frequencies of microtubules were measured by Sahu et al. [46] in the range of 10–30 MHz and 100–200 MHz. The resonant frequencies were disclosed by measurement of DC conductivity after application of oscillating signal of corresponding frequency and from transmittance and reflectance of microtubule without and with compensation of parasitic reactances of contacts in the frequency range from 1 kHz to 20 GHz. Transmission of the oscillating signals is independent of the length of microtubule. At the resonant frequencies, a sharp increase of DC conductivity was observed. At the particular frequencies, the transmittance is large and the microtubule resistance is much less than 0.04 Ω. Microtubule oscillators have a high quality factor. The peaks of resonance are not observed after release of water from the microtubule cavity. It should be mentioned that microtubule is also a multilevel memory. Electric current can store and erase 500 discrete bits in a single microtubule [47].

This paper contains an overview of the cancer initiation process as a pathological state of a complex biological system. The cancer transformation in the complex system contains biochemical-genetic links on the one hand and biophysical links on the other hand. The most important mechanisms involved in these processes concern cooperation of mitochondria and microtubules in the generation of the cellular electromagnetic field and production of force effects. We believe that gaining a biophysical understanding of the complexity of cancer processes may significantly contribute to improved cancer diagnostics and treatment.

## 2. Mitochondria Form Conditions for Microtubule Oscillations 

Production of ATP and GTP (adenosine and guanosine triphosphate) and triggering apoptosis are not the sole functions of mitochondria. The role of mitochondria in a cell is rather complex. Mitochondria and microtubules form a unique cooperating system in the cell [41, 48, 49]. Mitochondria alter the medium around them by the mechanism of proton transfer. Energy of pyruvate and fatty acids is used for pumping protons into the intermembrane space and in this way it is transformed into electrochemical proton gradient energy. From the intermembrane space, protons diffuse into cytosol through the outer membrane pores which are freely permeable to molecules whose relative molecular mass is 5,000 daltons or less. A layer of ordered water and a strong static electric field are formed around each properly working mitochondrion. Measurement of the intensity of the static electric field was performed by solid fluorescent particles of 30 nm in diameter [30]. At the outer mitochondrial membrane, the greatest intensity of the electric field (of about 3.5 MV/m) was measured. In the vicinity of a single mitochondrion, intensity of the electric field decreases nearly linearly as a function of distance. Even at a distance of 2 *μ*m from a mitochondrion, significant values of the electric field were measured (about 540 kV/m). This dependence may correspond to an ordered layer of water around a mitochondrion [29]. Water ordering is a phenomenon involving a change of the water structure from a viscosity liquid to a quasielastic gel affecting inner cellular processes, in particular providing low damping of the cytoskeleton vibration system. More than 20% of the cellular volume is occupied by mitochondria and the ordered water fills up most of the rest. Cytosol, cytoskeleton, and biological molecules are exposed to a strong electric field. Production of ATP utilizes the electrochemical proton gradient across the inner membrane. ATP is produced with efficiency higher than 40%. The rest of the nonutilized energy (nearly 60%) is liberated from mitochondria as heat, photons (emission of UV photons was detected too), and chemical energy not exploited for ATP and GTP production.

Localization of mitochondria depends on loci of energy consumption. In the interphase, mitochondria are concentrated around microtubules which are the organizing structures of the cytoskeleton. It is well known that microtubules consume energy in the form of GTP molecules required for polymerization by assembly competent tubulin dimers. In the M phase, the distribution of mitochondria is not precisely known. Microtubules are composed of tubulin heterodimers which carry large electric dipoles. Their oscillations generate an electromagnetic field (the near field whose great energy has characteristic features of the electric field is called electrodynamic field or the virtual photon field; at a greater distance from the source the electromagnetic character is dominating). The strong static electric field around mitochondria can shift microtubule oscillations into a highly nonlinear region.

The electrodynamic field generated by microtubules in the cellular cytoskeleton during different phases of the cell cycle has to be excited from the cellular energy sources. Energy supply is an essential condition for oscillations and generation of the electrodynamic field. Microtubules are dynamic cylindrically shaped polymers that display a so-called dynamic instability process with periods of growth interspersed with rapid shortening called a catastrophe. Energy supply to microtubules is provided by hydrolysis of GTP to GDP (guanosine diphosphate) in the ß tubulin unit of a heterodimer after the heterodimer is polymerized into a microtubule. Motor proteins transporting “cargo” move along microtubules using ATP molecules as energy sources. A part of the energy of motion is transferred to the microtubules (but the motor proteins might also cause disturbances of coherence and damping). In the interphase the greatest energy supply to microtubules is very likely provided by nonutilized energy liberated from mitochondria. In the M phase energy is supplied by treadmilling—polymerization from one end and depolymerization from the other end of microtubules in the mitotic spindle.

We argue that biological cellular activity depends on the generated electrodynamic field. Its role in the directional transport of mass particles and electrons [50, 51], organization of living matter [52], interactions between systems [53], and information transfer [8] was extensively analyzed and described. These works represent a new contribution to our understanding of the biological activity of living cells.

**Figure 1:**
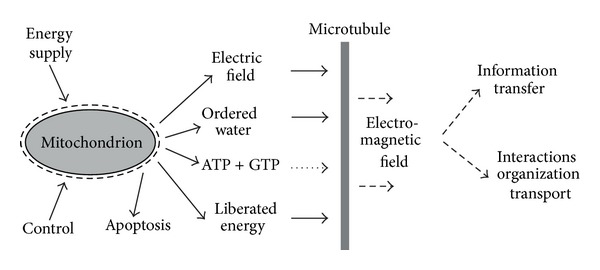
Biophysical mechanisms of biological activity of living cells depend on cooperation of mitochondria and microtubules. Mitochondrial function in healthy cells depends on transfer of protons from the matrix space into intermembrane space and to cytosol. Proton transfer is connected with formation of a strong static electric field and high level of water ordering in the mitochondrial neighborhood. Consequently, microtubule oscillations are strongly nonlinear and their damping is low. Microtubule oscillations are excited by supply of energy produced by mitochondria. Microtubules are electrically polar structures whose oscillations generate electrodynamic field which may participate in organization, transport of molecules and particles, interactions, and information transfer. Mitochondria function is disturbed in cancers. Inhibition of the pyruvate pathway in mitochondrion [54] results in partial suppression of proton transfer from the matrix space (nevertheless, diminished proton transfer may be caused also by other disturbances, for instance, in the citric acid cycle). Mitochondrial dysfunction causes lowering of static electric field and water ordering. Cancer cells with blocked pyruvate pathway (i.e., glycolytic phenotype cells) form a large group of cancers. The other large group of cancers has dysfunctional mitochondria in fibroblasts associated with cancer cells.

Figure 1 shows a schematic picture of the mitochondrion and microtubule activity and their cooperation. Mitochondria form conditions for coherent excitation of microtubules by energy supply, low damping, and shift of oscillations into a highly nonlinear region.

## 3. Biophysical Processes Disturbed by Cancer

Warburg assumed that partial suppression of the oxidative production of ATP and its replacement by fermentative (glycolytic) processes diminishes functional (and possibly structural) order in the cell. He commented on it stating that “the adenosine triphosphate synthesized by respiration therefore involves more structure than adenosine triphosphate synthesized by fermentation” [2]. Mitochondrial dysfunction disturbs all consequent physical processes and biological activity dependent on mitochondria. In healthy cells the oxidative energy production may be up to 100 times greater than the fermentative one (for instance, in kidney and liver cells). In cancer cells, only about one half of the ATP cell production is provided by mitochondrial supply. One type of mitochondrial dysfunction (called the glycolytic phenotype) is caused by inhibition of the pyruvate pathway by PDK—pyruvate dehydrogenase kinase [54]. Mitochondrial dysfunction was found in many types of cancer [55, 56]. In this connection, we stress the following facts: (a) a diverse group of information channels and oncogenes results in mitochondrial dysfunction with increased glycolysis and resistance to apoptosis, (b) the majority of carcinomas have so-called hyperpolarization of the mitochondrial inner membrane, and (c) most solid tumors exhibit an increased glucose uptake. These properties prompted Michelakis et al. to advocate targeting mitochondria in cancer treatments which may be effective in a large number of diverse malignant tumors, in particular using DCA (dichloroacetate) [57].

Potential of the inner membrane is an essential parameter for assessment of mitochondrial function. The potential is measured by uptake and retention of positively charged fluorescent dye, such as Rhodamine 123. Large uptake and retention is termed hyperpolarization. However, the uptake and retention may depend also on distribution of ions (for instance, K^+^) in the cell, production of lactate, and water ordering level and need not strictly correspond to the real mitochondrial inner membrane potential. The lack of mitochondrial hyperpolarization in certain types of malignant tumors, including oat cells lung cancer, lymphomas, neuroblastomas, sarcomas, and some other cancers [57, 58], suggests either a modified glycolytic phenotype or existence of another type(s) of mitochondrial defects and apoptosis blocking. By an electrically neutral exchange of protons and potassium ions, the pH gradient decreases and the membrane potential increases [58]. Defects in the mitochondrial respiratory enzyme complexes and electron carriers in the mitochondrial inner membrane might also diminish the proton transfer resulting in mitochondrial dysfunction in cancer cells. However, another deviation develops in cancerous tissue. Mitochondrial dysfunction is formed in fibroblasts associated with the cancer cell with fully active mitochondria—the reverse Warburg effect [59–62]. Energy rich metabolites (lactate, glutamine, etc.) are transported from the fibroblasts to the cancer cell. The state of enhanced mitochondrial energy production and activity may correspond to the lack of hyperpolarization. Therefore, the method of uptake and retention of a fluorescent dye can distinguish two different cancer mechanisms.

**Figure 2:**
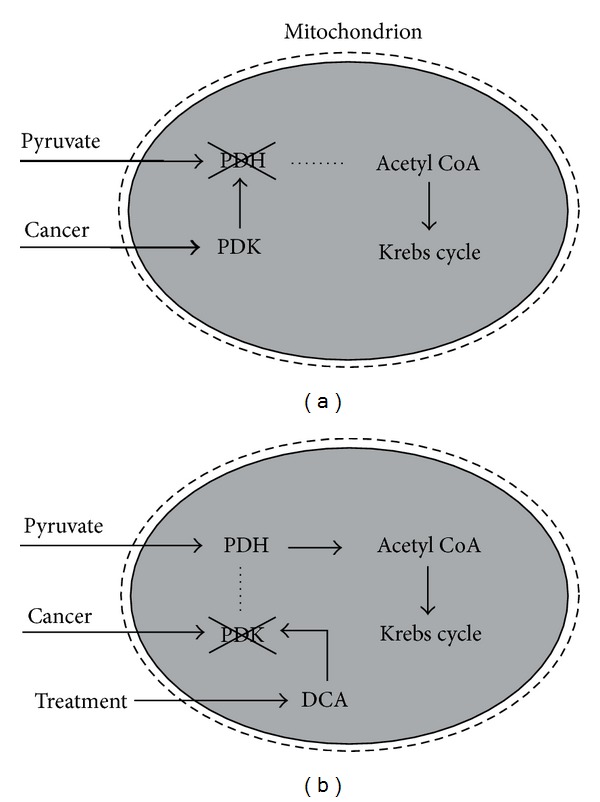
Glycolytic phenotype cancer cell may be treated by DCA (up to now the only known drug capable of restoring normal function of cancer cells). (a) The pyruvate pathway into the mitochondrial matrix space is blocked. PDH (pyruvate dehydrogenase) enzymes in the mitochondrial matrix (the grey area) phosphorylated by kinase PDK are dysfunctional, and pyruvate is not transferred to be broken down into the two-carbon acetyl groups on acetyl CoA (Coenzyme A). (b) DCA inhibits activity of PDK. Function of PDH enzymes is restored, and the pyruvate pathway in mitochondria is open (after [54]). The cell needs sufficient amount of oxygen for normal function.

Dependence of life processes on the real mitochondrial membrane potential may suggest its possible promotion of both life and death [63]. Basic processes of life are affected by potential disturbances caused by insufficient energy supply from pyruvate or fatty acids. Activity of pyruvate dehydrogenase (PDH) enzymes is regulated by PDH kinases (PDK-1-PDK-4). Mitochondrial dysfunction in the glycolytic phenotype cancer cell is caused by blocking the pyruvate pathway by the PDH kinases (see Figure 2). Hyperpolarization is accompanied by a low level of water ordering, diminution of the intensity of the static electric field around a mitochondrion, decrease of the nonutilized energy efflux, and low expression of the K^+^ channels. Importantly, DCA disturbs PDK-1, -2, and -4 [64] and in this way restores a normal mitochondrial activity resulting in normal cell function or switching on apoptosis of aberrant cells. Hyperpolarization is always associated with increased resistance to apoptosis [54]. A need for developing better PDK inhibitors than DCA was suggested [65]. It should also be mentioned that DCA action is based on attacking PDK and not on the mechanism of its production which could lead to the development of different pharmacological agents in the future.

Mitochondrial function is controlled by chemical and genetic signaling. But the altered mitochondrial function changes physical conditions in the cell affecting microtubule oscillations. As a final result, physical processes in the cell are altered, in particular mechanisms dependent on the electromagnetic field. Organization, transport, interactions, and information transfer are examples of such processes which are liable to be strongly disturbed. Consequently, the whole complex of the system activity exhibits disturbed behavior.

## 4. Cytoskeleton Filaments 

Actin filaments, microtubules, and intermediate filaments form a three-dimensional network providing a mechanical integrity of a cell. This network is collectively referred to as a cytoskeleton. Actin and tubulin proteins in their respective filaments bind a large number of different proteins, for example, ARP and MAP proteins, to enable participation in different functions in the cells. Microtubules form highly dynamic structures organizing the cell and generating electrodynamic fields around them. Cellular mechanical properties, dynamical behavior through the cell cycle including transport, and biological activity may strongly depend on the cytoskeleton organization and also on the generated electrodynamic field, in particular on its intensity, frequency spectrum, coherence, and spatial distribution pattern. The space pattern of the generated field is determined by geometrical arrangement of microtubules and other cytoskeleton structures. Cytoskeleton disturbances are presumably induced along the pathway of cancer cell development before malignant properties are fully established. Mechanical properties of healthy and cancer cells of the same tissue (investigated under action of external forces) are significantly different [66, 67]. Deformability of different cells of human origin was measured. Human nontumorigenic epithelial breast cells (MCF-10), nonmetastatic adenocarcinoma cells (MCF-7), and increased metastatic potential cells (modMCF-7) have different deformabilities 10%, 20%, and 30%, respectively [68]. Mechanical properties of human pancreatic cells (Pac-1) are altered after application of SPC (sphingosylphosphorylcholine) that plays a critical role in the metastatic invasion of gastrointestinal cancers. The keratin network shrinks around the nucleus, elasticity of the cell is reduced, and energy dissipated by mechanical deformation increased [69–71]. These effects might be caused by diminished electrodynamic interactions that are long range in comparison with chemical bond-making forces and biophysical contact interactions (a generated electromagnetic field may mediate interactions at a distance greater than 0.1 micrometer).

Some morphological changes used for cytological and histological evaluation of cancer development may result from cytoskeleton defects. For instance, in the cytological pictures, the keratin network shrinkage may be characterized by wrinkling of the nuclear membrane and disturbances of chromatin regular distribution—coarse chromatin clumping. Mitochondrial dysfunction may result in a lower intensity, lack of coherence, and a spatially diffused pattern of the electrodynamic field generated by microtubules in cancer cells of glycolytic phenotype in comparison with normal cells. Interaction forces between cells depend on the power and coherence of the generated electrodynamic field and due to the microtubule spatial organization on its spatial distribution pattern too. Interaction forces between cancer cells may be smaller than those between normal cells or between a normal and a cancer cell. For instance, cancer cells might be attracted by the normal cells around the tumor and pulled into healthy tissue. This force effect may constitute an essential part of the local invasion of the healthy tissue by malignant cancer cells [53].

Shrinkage of phosphorylated keratin filaments around the nucleus in response to SPC treatment precedes metastatic processes [69–71]. Due to the cytoskeleton disorganization, the space pattern of the generated electrodynamic field may be damaged to such an extent that the cancer cell can release itself from interactions with surrounding cells, liberate, and make metastases in distant organs. This process is well described in the cancer research literature and referred to as the epithelial-to-mesenchymal transition. However, we propose that it can be connected with a further decrease of the electromagnetic field intensity, level of coherence, and nonlinear properties of microtubules and a disturbance of the frequency spectrum. These mechanisms may be closely connected with the extracellular matrix defects which are known to be associated with the initiation of cancer.

Enslavement of cells in a tissue is assumed to depend on the generated electrodynamic field. The cells are identical and have the same conditions for generation of the electromagnetic field. Without essential changes the cell cannot evade enslavement and start independent activity in the body. On top of this, the changed cell has to escape from the supervision by the immune system. The region of tolerance of the electromagnetic field changes should be determined in order to elucidate this effect.

## 5. Treatment of Mitochondria in Cancer Patients

DCA is the first known drug capable of restoring normal healthy activity of a large variety of cancer cells. Regardless of this fact, its application to cancer treatment has been rare. Very likely the greatest clinical experience has been accumulated in the Medicor Cancer Centres in Canada where patients with advanced stages of cancer are treated by DCA; casual reports are available ([72]; http://www.medicorcancer.com/). A wide spectrum of various tumors has been treated. For instance, metastatic renal, lung, and ovarian carcinoma, mesothelioma, glioblastoma, and melanoma with brain metastasis were reported. The minimum and the maximum doses were 10 and 25–50 mg/kg/day, respectively. The courses were continual or cyclic (1–3 weeks on followed by 1 week off). Duration of the treatment should be at least one month. Doses are limited by severity of the side effects. For doses equal to or greater than about 20 mg/kg/day, the response is developed within 2–4 weeks. For smaller doses the response is weaker or delayed. A positive response was reported to have been experienced by 60%–70% of treated patients.

Side effects are claimed to be mild and reversible in the majority of cases ([72]; http://www.medicorcancer.com/). Description of the side effects is also in an overview on mitochondrial targeting for cancer treatment [73]. The side effects are dose and age dependent. In some experimental animals dichloroacetic acid may induce liver cancer by long-term exposure at doses of 100–1000 mg/kg/day. However, in humans a short-term DCA administration appears to be relatively nontoxic. Exposure to low doses of about 25 mg/kg/day for several months did not reveal adverse side effects [74]. The side effects observed so far are of neurological and gastrointestinal origin. Neurological side effects concern peripheral neuropathy, sedation, fatigue, confusion, hallucination, memory problems, hand tremor, and gait disturbances. Gastrointestinal side effects include heartburn, nausea, vomiting, and indigestion. The most dangerous process involved is the tumor lysogenic syndrome. If a large number of cells is decomposed by apoptosis in a short time, then a sudden release of the dead cell material into the blood stream may cause abnormal heart rhythms and kidney failure.

DCA has antitumor effects, relatively low toxicity (however, dependent on the dose and period of administration), and low cost. In the positively responded patients, DCA offers a palliative effect. In these “palliative” patients, DCA appears to demonstrably improve the quality of life. However, it can also transform advanced stages of cancer from a fatal disease to a chronic disease treatable with simple medications. Adjuvant DCA can occasionally cure stage 4 cancer. Cancer treatment provided in the Medicor Cancer Centres is based on a combination of DCA treatment with chemotherapy, radiotherapy, and surgery. The most effective combination of drugs for each individual tumor is found by the chemosensitiveness test. Medicor Cancer Centres announced that they had treated about 1300 patients. All these patients were previously treated by standard methods and capabilities of conventional cancer therapies which came to an end—the treatment was either ineffective or could not continue. Most of the patients were in advanced stages of the disease. As expected, DCA appears to be more effective in healthier patients as opposed to patients with a very advanced disease. Therefore, response to DCA in patients with an initial disease stage may be more promising.

## 6. Discussion

Excitation of electromagnetic fields in living cells is one of the essential parts of the biophysical processes taking place at subcellular levels. Its generation in living cells was claimed to be impossible due to water viscosity damping [75] or insufficient energy sources for excitation of oscillations in the cell [76, 77]. However, the former authors neglected to consider water ordering and the latter authors did not take into consideration high quality factor of biological oscillators and the key nonlinear properties of the cellular system.

Cellular electromagnetic fields are generated by microtubules. Cooperation of microtubules with mitochondria plays an essential role in living cells leading to the establishment of a functional level of biophysical processes. Generation of electromagnetic fields by microtubules in living cells crucially depends on the function of mitochondria. Mitochondria are regulated by chemical-genetic signaling, but besides triggering apoptosis their activity is mainly connected with physical mechanisms. Mitochondrial function cannot be reduced to energy conversion into ATP and GTP. Transfer of protons from the matrix space into cytosol creates strong static electric fields around mitochondria with consequences that include nonlinear effects on microtubules and water ordering in the cytosol. Mitochondria perform an essential role in cell organization and cell activity in general. Their dysfunction disturbs biophysical processes. This is the case in the vast majority of cancers. At a certain stage of cancer development, mitochondrial dysfunction is formed and affects numerous properties of cells including spatial organization and functional order. Chemical, genetic, and physical mechanisms are mutually coupled.

Diversity of cancer origin agents also led to a hypothesis that mitochondrial dysfunction is a primary cause of cancer and biochemical and genetic deviations develop as consequent events [78]. This hypothesis has not been proved yet and some inconsistency with experimental results may be found. In cervical cancer cells formation of the mitochondrial dysfunction is observed in the time period of the development from precancerous lesions to cancer cells (measured by the immune system response to LDH virus antigen and specific tumor antigen—Jandová et al. [79]), that is, after biochemical and genetic changes. Mitochondrial dysfunction is a result of chemical-genetic defects (on the other hand dysfunction of mitochondria might be caused by a specific agent directly without any previous changes in the biochemical-genetic region). Mitochondria are the boundary entities between chemical-genetic and biophysical processes. Mitochondrial dysfunction disturbs essential biophysical processes in living cells [41, 48, 49]. However, it should be kept in mind that there is an exceptional cancer type characterized by normally functioning mitochondria. Pavlides et al. [59] describe a type of cancer, where energy rich metabolites are transported to cancer cells for utilization in efficient mitochondrial production. These cancer cells with highly active mitochondria display increased malignity which may correspond to enhanced electrodynamic excitation.

Disturbances of biophysical processes might be also-caused by defects in the link to the generation of the electromagnetic fields. For instance, asbestos carcinogenicity is hypothetically explained as having a capability to lower the electrodynamic activity in cells by forming transmission fibers which short-circuit distant parts of the cell with different levels of the electromagnetic field [80]. If it is so, asbestos transformation pathway begins beyond the mitochondrial link and disturbs directly the electrodynamic activity of the cell. Asbestos carcinogenicity has been explained on the basis of several different mechanisms including oxidative stress, chromosome tangling, or adsorption of specific proteins and carcinogenic molecules which may contain iron atoms (Toyokuni, [81]). Toyokuni also explained the carcinogenicity of iron as an effect of reactive oxygen species—ROS [82]. But metal particles might disturb the electromagnetic activity of the cell by a mechanism similar to the short-circuits created by asbestos. The iron carcinogenicity needs not be only a result of ROS but also of the short-circuit effects. Therefore, this raises a question whether treatment based on drugs increasing electric conductivity might cause adverse effects in the afflicted normal cells.

Nevertheless, mitochondrial dysfunction seems to be the most common defect in cancers disturbing their biophysical and consequently the biological behavior. Discovery of electromagnetic activity in living cells may improve our understanding of biological activity and its disordering by cancer. Microtubule oscillation frequencies are one of the fundamental parameters required to be determined in this connection. Nanotechnological sensors and amplifiers may be used for measurement of electrodynamic activity of healthy and cancer cells in the frequency range from about 1 MHz to 1 GHz to determine physical differences (nevertheless, electrodynamic activity in kHz range is reported too). The resonant frequency may depend on excitation due to nonlinear nature of oscillations in microtubules. If the frequency is determined by the secondary structure of tubulin, then proteins should be able to oscillate at resonant frequencies and electrically polar protein molecules generate electrodynamic fields. Electrodynamic fields are also generated by rotation and rotation-vibrational motion of electrically polar molecules and organized structures. Based on these considerations long-range interactions between individual proteins are likely to not only exist but to play important roles in living cells. Therefore, drugs interacting attractively with a convenient target can be synthesized. This supports the idea of preparation of carrier or helper particles that would transport molecules of chemotherapeutic drugs and direct them at the predetermined targets. Efficacy of treatment would be enhanced and side effects diminished.

A cancer transformation pathway is formed by a complex microevolution, multistep, and multibranched process. Essential life mechanisms are misused and gradually altered by cancer. The complex biological system is deformed. Adaptability of cancer cells to environmental changes and diverse cellular stresses is high, arguably higher than in normal cells. Any “one-point” treatment may be overcome by altered cancer mechanism. Adaptability and heterogeneity of cancer processes is an obstacle in their efficient treatment. Therefore, the treatment should be complex, targeting essential links along the cancer transformation pathway and reproducible in recurrent cases. Dysfunctional mitochondria are such an essential link that cannot be bypassed by altered cancer mechanism. Moreover, differences between healthy and cancer cells used for treatment seem to be very often of quantitative type. Treatment of malignant tumors based on the therapeutic strategy of killing the tumor cells almost always negatively affects the healthy cells, in particular those that proliferate rapidly, such as epithelial cells, blood cells, and the immune system. The negative side effects may be individual and vary from negligible to serious. For instance, the differences in increased fermentative ATP production levels may belong to a quantitative type. Cancer therapy based on inhibiting fermentative energy production may considerably damage healthy cells too.

Cancer treatment should target the processes and structures that exhibit the most significant deviations from a normal physiological state. Dysfunction of mitochondria is a very remarkable difference between a healthy and a cancer cell of the glycolytic phenotype. As a result of mitochondrial dysfunction (due to diminished static electric field and water ordering), the endogenous electrodynamic field generated in cancer cells has a decreased intensity, coherence, disturbed frequency spectrum, and spatial pattern. Except for some cases mentioned earlier, mitochondrial dysfunction represents the greatest functional differences in comparison with healthy cells [41, 48, 49]. Restoration of normal mitochondrial function reestablishes conditions for normal physical processes and unlocks the apoptotic pathway. If the cell is too aberrant, for instance, by disorganization of the cytoskeleton or the DNA structure, mitochondria can send a signal to start the preprogrammed cell death (apoptosis). Targeting mitochondria very likely acts on the region of essential differences between healthy and cancer cells. It may be assumed that an effective therapeutic strategy of cancer treatment should aim at a restoration of normal mitochondrial function. In the case of the reverse Warburg effect cancer cells are highly excited due to supply of energy rich metabolites. The normal mitochondrial function should be restored in associated fibroblast and the transfer of energy rich metabolites to cancer cells cut off.

Defects causing mitochondrial dysfunction may form the main target of cancer therapy. Inhibition of the pyruvate pathway into mitochondria is a well-known defect. Exchange of protons for potassium ions in the transfer across the inner membrane may disturb mitochondrial function too [58]. But other types of defects may also exist, for example, inhibition of the electron pathway of the oxidation cycle in the inner mitochondrial membrane. The only known effective drug for restoration of the pyruvate pathway and normal mitochondrial function in a large group of cancers is DCA. This drug inhibits some PDK (-1, -2, -4) blocking pyruvate transfer and its utilization in the mitochondrial matrix. Some other drugs (such as vitamin E analogs [83, 84]) targeted at mitochondria kill cells through destabilization of mitochondria and induction of apoptosis. Nevertheless, the killing process needs not be limited only to cancer cells and could also inflict negative effects on healthy cells.

A considerable amount of preclinical evidence of DCA effects *in vitro *and *in vivo *has already been accumulated. Supporting experience in human cancer treatment is substantial too. The treatment effects in Medicor Research Centres were mainly palliative. However, it should be noted that mainly advanced stage cancer patients were treated there. Clinical trials with less advanced cancer patients should be started to bring further confirmation of positive effects in DCA cancer treatment. Observations of tumor reaction and development should be performed together with examinations of patient states based on laboratory tests, measurements, clinical findings, and analysis of specific symptoms after DCA application. Besides, many essential issues of DCA application have remained unknown, in particular, whether the treated cells had sufficient amount of oxygen to start normal mitochondrial function. Important points may also concern the type of chemical reaction provided by DCA, its target loci at the PDK's and other structures, and possible changes caused by DCA in its target molecules or structures. Some compounds containing sodium, chlorine, and oxygen elements might be more effective than DCA, especially those whose conformation of oxygen atoms is similar to that in DCA (such as chlorine dioxide). It is known that sodium chloride induces necrosis of ovarian carcinoma cells and hypochlorous acid enhances immunogenicity by activation of tumor-specific cytotoxic T cells [85]. Clinical trials with DCA and examination of DCA reactions in the cell may determine specific requirements for future drug development and open a way for a new strategy in cancer treatment, restoring the normal function of mitochondria, the whole cell, and unlocking apoptosis, depriving cancer cells of their immortality which is the main proliferative advantage over normal cells.

## 7. Conclusions

Coherent electromagnetic fields generated by microtubules in living cells represent a new and outstanding issue in present-day cell biology. Effectiveness of microtubule oscillations depends on mitochondrial function. Besides ATP and GTP production and liberation of nonutilized energy mitochondria form important boundary links between chemical-genetic and physical processes in living cells. They set up conditions for physical mechanisms in living cells. Establishment of a strong static electric field and formation of a layer of ordered water around mitochondria belong to essential conditions for the generation of coherent electrodynamic field by microtubules. The electrodynamic field can provide directional transport in the cell, facilitate organization of structures and organelles, and affect interactions in the cell and between cells, including information transfer.

Inhibition of the pyruvate transfer into mitochondrial matrix causes mitochondrial dysfunction in a large group of cancers. Other defects (for instance, in the citric acid cycle) diminish proton transfer from the matrix space too. Dysfunction of mitochondria results in decreased static electric field, diminished water ordering around them, and lowered energy supply. Consequently, electrodynamic field generated by microtubules is characterized by low power, diminished coherence, and altered frequency spectrum. The space pattern of the field may be disordered too. Biological functions dependent on generated electrodynamic fields are very likely to be disturbed as a result.

However, there is another group of cancers. Mitochondrial dysfunction is not developed in a cancer cell but in the associated fibroblasts. The supply of energy rich metabolites from associated fibroblasts results in a strong aggressiveness of cancer cells. This cancer type deserves special analysis concerning increased electrodynamic activity.

We propose that essential and specific differences between healthy and cancer cells should be exploited for the development of a new generation of cancer therapies. The conventional cancer therapeutic strategy is based on cancer cell killing with associated collateral damage to healthy tissue. The main effort is aimed at finding sufficiently specific property to kill the cancer cells and to limit damage to healthy cells. However, the healthy cells may be damaged too and treatment of recurrent cancers remains unsolved.

Mitochondrial dysfunction may be a specific and essential difference between healthy and cancer tissues. One cause of the dysfunction is known—inhibition of the pyruvate pathway into mitochondrial matrix. Treatment by DCA of cancers with the Warburg effect and by DCA in combination with glycolysis inhibitors of cancers with the reverse Warburg effect can stimulate synthesis of new drugs restoring physiological role of mitochondria and normal function of various cancer cells.
